# Differentiating SGBS adipocytes respond to PPARγ stimulation, irisin and BMP7 by functional browning and beige characteristics

**DOI:** 10.1038/s41598-019-42256-0

**Published:** 2019-04-09

**Authors:** Ágnes Klusóczki, Zoltán Veréb, Attila Vámos, Pamela Fischer-Posovszky, Martin Wabitsch, Zsolt Bacso, László Fésüs, Endre Kristóf

**Affiliations:** 1 Department of Biochemistry and Molecular Biology, Faculty of Medicine, University of Debrecen, Debrecen, Hungary; 2 Regenerative Medicine and Cellular Pharmacology Research Laboratory, Department of Dermatology and Allergology, University of Szeged, Szeged, Hungary; 3 Division of Pediatric Endocrinology and Diabetes, University Medical Center Ulm, Ulm, Germany; 4 Department of Biophysics and Cell Biology, Faculties of Medicine and Pharmacology, University of Debrecen, Debrecen, Hungary

## Abstract

Brown and beige adipocytes are enriched in mitochondria with uncoupling protein-1 (UCP1) to generate heat instead of ATP contributing to healthy energy balance. There are few human cellular models to reveal regulatory networks in adipocyte browning and key targets for enhancing thermogenesis in obesity. The Simpson-Golabi-Behmel syndrome (SGBS) preadipocyte line has been a useful tool to study human adipocyte biology. Here we report that SGBS cells, which are comparable to subcutaneous adipose-derived stem cells, carry an FTO risk allele. Upon sustained PPARγ stimulation or irisin (a myokine released in response to exercise) treatment, SGBS cells differentiated into beige adipocytes exhibiting multilocular lipid droplets, high UCP1 content with induction of typical browning genes (*Cidea, Elovl3*) and the beige marker *Tbx1*. The autocrine mediator BMP7 led to moderate browning with the upregulation of the classical brown marker *Zic1* instead of *Tbx1*. Thermogenesis potential resulted from PPARγ stimulation, irisin and BMP7 can be activated in UCP1-dependent and the beige specific, creatine phosphate cycle mediated way. The beige phenotype, maintained under long-term (28 days) conditions, was partially reversed by withdrawal of PPARγ ligand. Thus, SGBS cells can serve as a cellular model for both white and sustainable beige adipocyte differentiation and function.

## Introduction

Studies focusing on the therapeutic potential of brown adipose tissue (BAT) against obesity have increased over the last decade^1^. Two types of thermogenic cells, the classical brown and beige/brite adipocytes^2,3^, are present in mammals. In mice, the classical brown adipocytes in BAT are constantly available in the interscapular area for heat production. The development and activity of beige adipocytes can be induced in white adipose tissue (WAT), as a result of cold^4^, ß3-adrenergic stimulation^3,5^ or long-term treatment with peroxisome proliferator-activated receptor γ (PPARγ) agonists^6^. Thermogenesis of both classical BAT and beige adipocytes is mediated by the mitochondrial uncoupling protein-1 (UCP1) located in the inner mitochondrial membrane^7^. In these cells, the energy generated by the respiratory chain in the form of the proton gradient is dissipated, by the assistance of elongated fatty acids. This results in heat production instead of ATP synthesis^8^.

Detailed studies in rodents revealed that, in spite of functional and morphological similarities, classical brown and beige adipocytes have distinct origins^9^. Brown adipocytes derive from the same precursors as myocytes^10^. On the other hand, white and beige adipocytes have different common mesenchymal precursors and their developmental programs are separated at a certain point of differentiation^11^. When adrenergic stimulation, leading to the appearance of beige adipocyte population marked by high *Tbx1* and *Cited1*^12^ expression, is eliminated, beige adipocytes can be reversed into masked beige (white looking) cells^13^. Genes, like zinc finger protein 1 (*Zic1*), have been described as classical brown adipocyte markers which are not expressed by the beige adipocytes^14^.

Irisin is a myokine, cleaved from the FNDC5 transmembrane protein, which acts as a browning-inducer endocrine hormone, through integrin α_V_β receptors^15^ and via the p38 MAPK and ERK pathways^16^. In mice, physical exercise as well as shivering induced the secretion of irisin by skeletal myocytes which resulted in a marked beige differentiation in the subcutaneous WAT^17^. The p38 MAPK signaling is also induced by distinct bone morphogenetic proteins (BMPs), including BMP7 which is an auto/paracrine mediator in mice that both drive classical brown and beige adipocyte differentiation^18,19^. Another key endocrine hormone, atrial natriuretic peptide (ANP), which is secreted by cardiomyocytes and switches on p38 MAPK signaling, promotes UCP1-dependent thermogenesis and mitochondrial biogenesis of murine and human beige adipocytes^20^. Besides UCP1-dependent thermogenesis, UCP1-independent heat-producing mechanisms were described as a beige specific feature^13^. One example is a creatine-phosphate futile cycle, which requires coupled ATP synthesis. During this process, creatine kinase catalyzes the phosphorylation of creatine using ATP generating phospho-creatine and ADP. Then, phospho-creatine is immediately dephosphorylated which results in heat production^21^. The importance of this cycle was demonstrated in mice which lack in adipose tissue the rate-limiting enzyme of creatine biosynthesis, glycine amidinotransferase developed diet-induced obesity due to the suppression of thermogenesis mediated mostly by beige adipocytes^22^.

In contrast to detailed studies in rodents, there are only limited data about regulatory networks that drive human brown or beige adipocyte differentiation and activation. In adult humans, brown adipocytes are localized to the deep neck, supraclavicular or perispinal regions^23,24^. However, these regions are difficult to access for sample collection. Human adipose-derived mesenchymal stem cells (hADMSCs) isolated from stromal vascular fractions (SVFs) of abdominal subcutaneous fat were able to differentiate into functional thermogenic adipocytes, presumed to be beige cells, in response to different stimuli^25–29^. The creatine-driven futile cycle was also active in human beige adipocytes of different anatomical origins^21,28^. However, when irisin was administered directly to differentiating human hADMSCs, inconsistent effects were observed^25–27,30^.

It has been recently clarified that SNP rs1421085 T-to-C variant underlies the genetic association between the FTO locus and obesity. The presence of the risk-allele of the FTO locus results in a cell autonomous shift in the gene expression programs toward white instead of beige adipocyte with lipid-storage and a decrease in thermogenesis in response to cold stimulus^31^. Of note, to our knowledge, the expression of the 2-oxoglutarate dependent dioxygenase enzyme encoded by the *Fto* gene is not influenced by the presence of the aforementioned risk-allele.

Human cell lines and cellular models are needed to explore more the key molecular elements of browning and targets of possible pharmacological treatments that can enhance browning. Shinoda *et al*. isolated SVF from supraclavicular and subcutaneous WAT from two healthy individuals, immortalized them, and found that brown adipocytes derived from single cell derived clones of preadipocytes from the supraclavicular regions displayed molecular signatures which resembled thermogenic beige adipocytes^32^. Xue *et al*.^33^ generated human preadipocyte cell populations derived from the supraclavicular region of four healthy patients and immortalized them. Using similarly generated preadipocyte clones, genes which may determine the commitment to differentiation to brown adipocytes in the supraclavicular area have been identified^33^. A non-immortalized human preadipocyte cell line, namely the Simpson-Golabi-Behmel syndrome (SGBS) cell strain was described by Wabitsch *et al*.^34^, in 2001 and it turned out to be a useful model for white adipocyte differentiation^35–38^. SGBS cells behave very similarly to human primary preadipocytes and the differentiated white SGBS adipocytes cannot be distinguished from human primary adipocytes by morphology or in functional terms after culturing them in adipogenic medium. Furthermore, it has been also demonstrated that knockdown of 2-oxoglutarate dependent dioxygenase (encoded by the proposed *Fto* gene)^39^ or teneurine-2 in SGBS preadipocytes^40^ using siRNA induced both UCP1 mRNA and protein expression upon adipogenic differentiation raising the possibility that SGBS cells represent a preadipocyte population with a significant beige potential. In the present study, we investigated systematically how browning of SGBS cells can be induced by PPARγ, irisin and BMP7 stimuli, and found that browning differentiation results in functional and sustainable beige cells.

## Results

### SGBS cells express surface markers similarly to primary preadipocytes and are heterozygous for the FTO risk allele rs1421085

Primarily, we examined undifferentiated SGBS cells by surface antigen expression analysis. We found that hematopoietic/monocyte markers (CD34, CD47), endothelial markers (CD54), fibroblast markers (CD73, CD90), integrins and CAMs (integrin ß1, CD44, CD325) were expressed on the surface of undifferentiated SGBS preadipocytes (Supplementary Fig. S1a). Then, we compared the surface antigen expression pattern of SGBS preadipocytes to SVF cells isolated from human abdominal subcutaneous fat^41^. Most of the investigated markers were expressed similarly in SGBS and primary preadipocytes. However, CD34, CD44, CD146 and HLA-DR expression levels were higher in SGBS preadipocytes, while CD105, CD49a and CD31 antigens were expressed at a lower level compared to primary preadipocytes (Supplementary Fig. S1a). Next, we tested the presence of the C risk-allele of the rs1421085 locus; DNA sequencing (Supplementary Fig. S1b) and qPCR-based genotyping analysis (data not shown) determined that SGBS cells are heterozygous for the C risk allele.

### SGBS preadipocytes respond to sustained PPARγ ligand and irisin or BMP7 treatment by inducing either beige or classical brown marker genes

**Figure 1:**
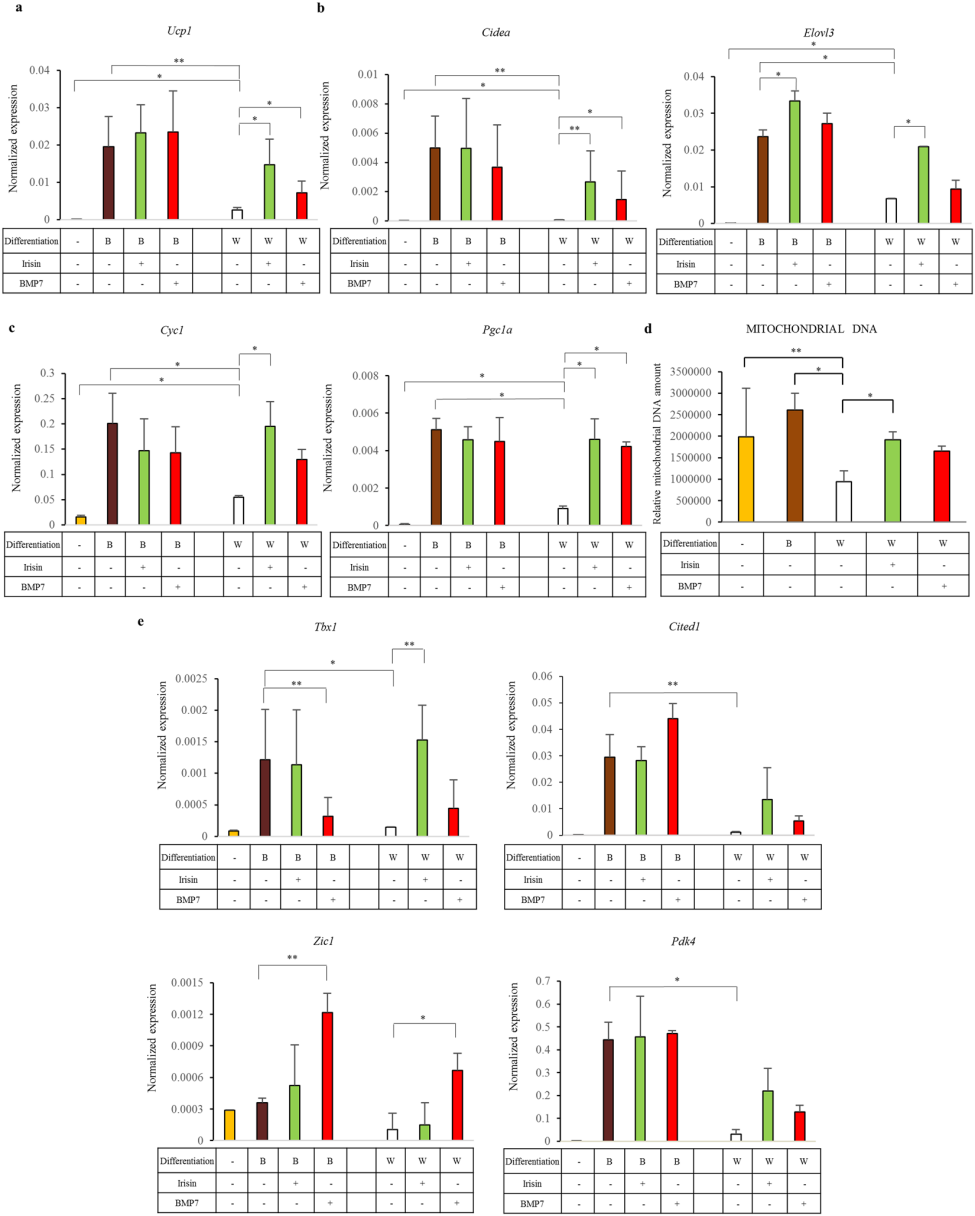
Browning of SGBS cells is induced by PPARγ-driven differentiation cocktail, irisin or BMP7 treatment. SGBS preadipocytes were differentiated to white (W) or brown (B) for two weeks; human recombinant irisin treatment at 250 ng/ml concentration (green bars) or BMP7 treatment at 50 ng/ml concentration (red bars) were applied to induce browning of SGBS cells from day 1. Expression of *Ucp1* **(a)**, *Cidea*, *Elovl3* **(b)**, *Cyc1* **(c)**, *Tbx1*, *Cited1*, *Zic1, Pdk4* **(e)** genes in SGBS adipocytes. Gene expression was determined by RT-qPCR and target genes were normalized to *Gapdh*. Relative mitochondrial DNA content of preadipocytes and differentiated SGBS cells determined by quantitative PCR **(d)** n = 5 *p < 0.05; **p < 0.01.

We applied previously described white (initiated by four days treatment with the PPARγ-ligand rosiglitazone)^36^ and browning (with the continuous presence of rosiglitazone during differentiation)^29^ protocols to differentiate SGBS preadipocytes and compared the expression of selected thermo- and adipogenic marker genes^27^ in the two settings. The browning cocktail highly induced *Ucp1* mRNA expression. Similarly, the presence of human recombinant irisin or BMP7 on the top of the white differentiation protocol resulted in enhanced *Ucp1* mRNA expression; presence of irisin or BMP7 in the browning cocktail did not increase *Ucp1* expression further (Fig. 1a). mRNA of brown-fat specific genes, like *Cidea* and *Elovl3* were also enriched during the administration of the browning cocktail and when irisin was added to the white differentiation cocktail (Fig. 1b). In contrast, we observed decreased expression of *Lep*, a white adipocyte marker gene, in response to the browning differentiation (the effect of irisin and BMP7 was not significant in that respect). The expression of other adipogenic markers, *Cebpb* and *Pparg*, did not differ between white and browned SGBS adipocytes. *Fabp4* was expressed at a significantly higher level in browned adipocytes compared to the white ones. Out of these markers, only the expression of *Pparg* was increased in response to irisin or BMP7 treatment (Supplementary Fig. S2).

The expression levels of the mitochondrial enrichment marker, *Cyc1* and the master regulator of mitochondrial biogenesis, *Pgc1a* were significantly higher in browned SGBS cells compared to white adipocytes and irisin treatment had the same effect (Fig. 1c). In the undifferentiated SGBS preadipocytes we could detect high mitochondrial DNA content. Differentiated white adipocytes have relatively lower mitochondrial DNA content and irisin treatment resulted in significantly elevated mitochondrial DNA amount in them while the effect of BMP7 was moderate. The mitochondrial DNA amount was the highest in the case of browned cells after the application of the PPARγ-driven browning differentiation cocktail (Fig. 1d).

Next, we asked the question whether the beige-selective marker genes, including *Tbx1*^42^ and *Cited1*^12^ or classical brown adipocyte markers, like *Zic1*^23^ were induced in SGBS cells. Irisin or the browning protocol resulted in marked upregulation of *Tbx1* and *Cited1* but no *Zic1* induction. There was no further increase of *Tbx1* and *Cited1* expression when irisin was added on top of the browning protocol. BMP7, on the other hand, only upregulated *Zic1* markedly and even prevented *Tbx1* induction when it was combined with the browning differentiation cocktail (Fig. 1e). The expression of *Tmem26* and *Shox2*, proposed beige-markers^43^ reported in mouse studies, was not elevated significantly in the browned SGBS adipocytes as compared to the white ones (Supplementary Fig. S3). Furthermore, *Tnfrsf9* (CD137) was expressed markedly only in undifferentiated preadipocytes (data not shown). Interestingly, *Pdk4*, originally described as a classical brown marker in mice^44^, showed a similar expression pattern as the beige-selective *Tbx1* and *Cited1*, in SGBS adipocytes (Fig. 1e).

Then, we determined the concentration dependence of the irisin and BMP7 effects during white adipocyte differentiation in SGBS cells. Jedrychowski *et al*.^45^ found that irisin was present at 3–4 ng/ml concentration in human blood plasma in the case of sedentary life but increased to 4–5 ng/ml if individuals took part in aerobic training. *Itgav*, the gene which encodes the specific subunit of the recently identified irisin receptor^15^ is expressed both in undifferentiated SGBS preadipocytes and in differentiated adipocytes to the same extent (Supplementary Fig. S3). At 5 ng/ml concentration of irisin only a slight increase could be observed in *Ucp1* expression compared to untreated cells. However, irisin was efficient above 50 ng/ml concentration. The same trend was observed when the expression of *Tbx1*, *Cyc1*, *Elovl3* and *Pgc1a* was analyzed (Supplementary Fig. S4). The supplementation of BMP7 to the white adipogenic differentiation media in increasing concentrations resulted in upregulated expression of *Ucp1* and *Cidea* brown marker genes compared to the untreated white adipocytes. In contrast, we observed downregulation of *Lep* at 100 ng/ml BMP7 concentration. However, we could not detect significant changes in the expression of *Tbx1*, *Cyc1*, and *Elovl3* as a result of BMP7 treatment (Supplementary Fig. S5).

**Figure 2:**
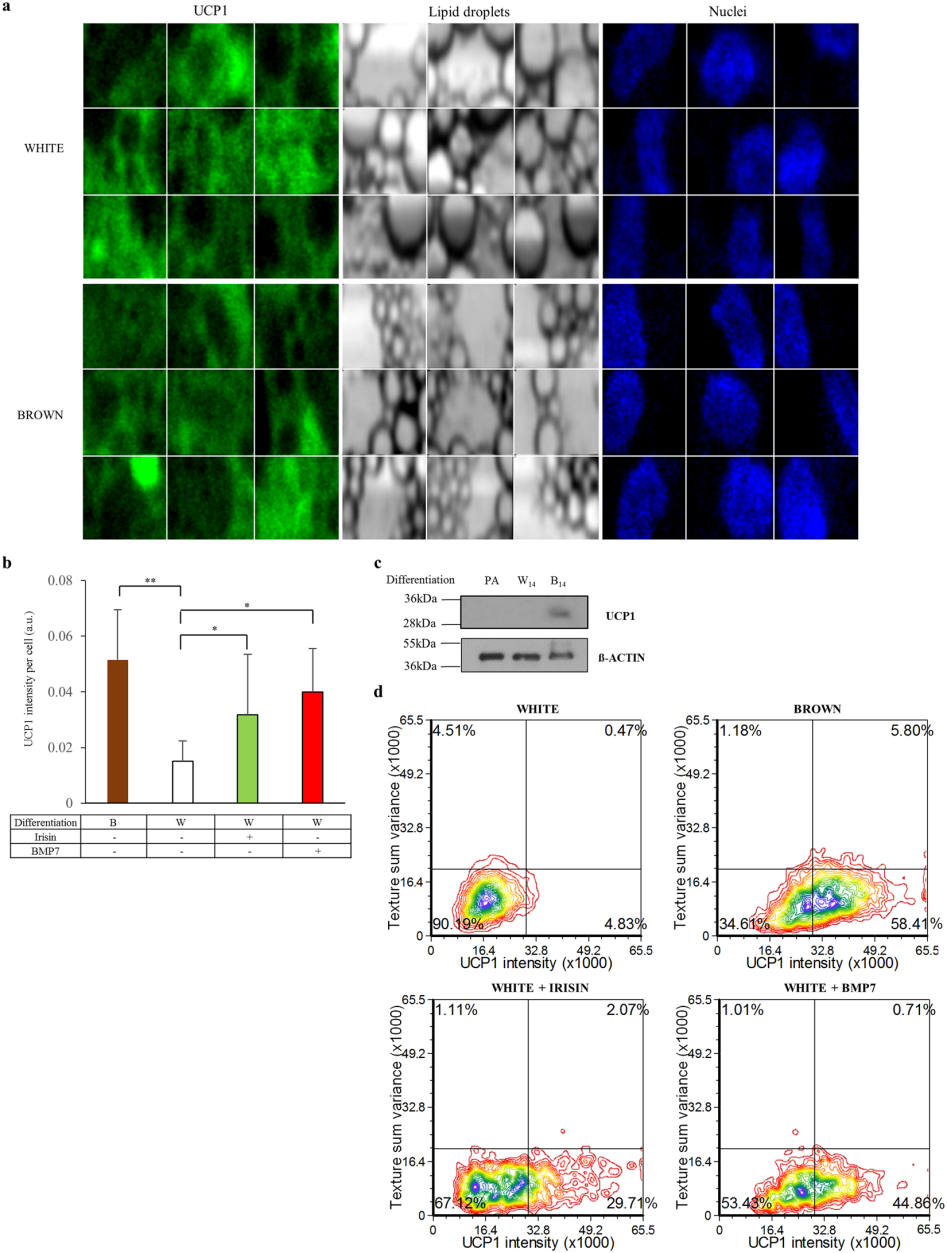
Morphological features of browning SGBS adipocytes that were induced by PPARγ-driven differentiation cocktail, irisin or BMP7 treatment. UCP1 content, lipid droplets and nuclei are shown in gallery images (N = 9) of selected cells within the white and brown differentiated adipocytes (see an overview image of a sample of brown differentiation in Supplementary Fig. S6). SGBS preadipocytes were differentiated and treated as in Fig. 1. Images of differentiated samples were captured by a laser-scanning cytometer in 3 independent experiments. In every experiment 1000–2000 cells per each sample were recorded and measured. Applying image analysis, single cells were identified based on their nuclei and classified according to their UCP1 content and lipid droplet structure. (**a**) UCP1 protein content of browning induced adipocytes (PPARγ-driven differentiation cocktail, irisin or BMP7 treatment) as compared to white adipocytes. (**b**) Expression of UCP1 at protein level in SGBS adipocyte lysates detected by immunoblotting. (**c**) The blot was cropped. The original picture of the full-length blot is displayed in Supplementary Fig. S7. Density plot figures show texture sum variance and UCP1 protein content of differentiated single cells in one representative SGBS replicate. (**d**) *p < 0.05; **p < 0.01.

As a next step, we investigated the morphological characteristics of the white and browned SGBS cells by assessing the textural parameters and UCP1 protein content of the individual adipocytes using laser-scanning cytometry (Fig. 2a, Supplementary Fig. S6). We found higher UCP1 content in single SGBS adipocytes (Fig. 2b) and in total cell lysates (Fig. 2c) as a result of the 14 day-long PPARγ-driven browning differentiation, as compared to white. Moreover, we detected increased UCP1 protein content in response to irisin or BMP7 administration (Fig. 2b). In contrast to primary adipocytes^27,46^ the texture sum variance did not change significantly during the induction of browning marked by higher UCP1 expression (Fig. 2d).

### Differentiated SGBS adipocytes respond to activation as functional beige cells using the creatine phosphate cycle

**Figure 3:**
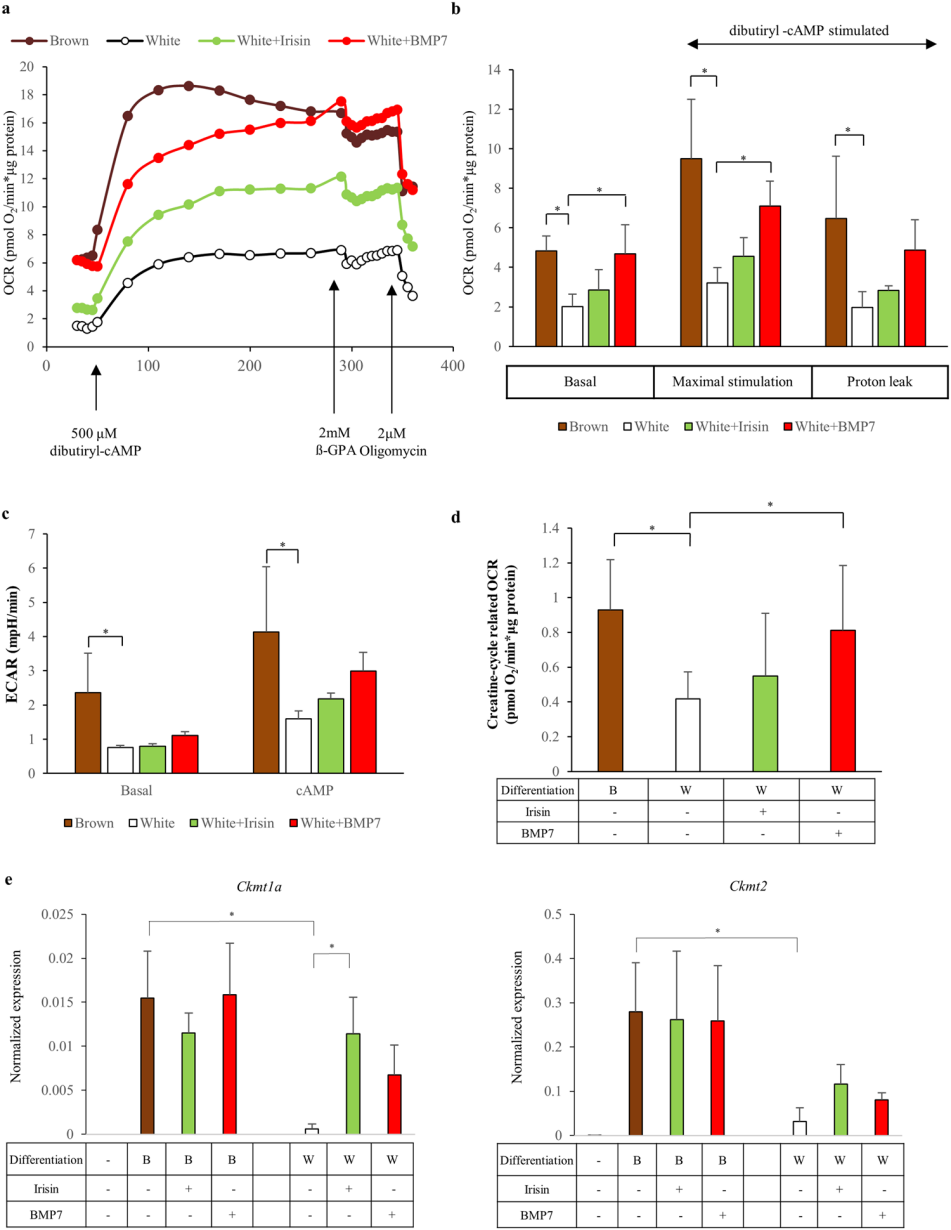
Functional measurements detect high oxygen consumption and significant involvement of the creatine substrate cycle in Rosiglitazone, irisin and BMP7 differentiated browning adipocytes. SGBS preadipocytes were differentiated and treated as in Fig. 1. Mitochondrial oxygen consumption rate (OCR) of differentiated SGBS cells of one representative measurement determined by a Seahorse XF96 analyzer. (**a**) Basal, dibutyryl-cAMP-stimulated and oligomycin-inhibited oxygen consumption levels in SGBS cells, compared to basal OCR of white-directed adipocytes. (**b**) Extracellular acidification rate (ECAR) of differentiated SGBS cells measured by a Seahorse XF96 analyzer. (**c**) Inhibitory effect of ß-guanidiopropionic acid (ß-GPA) (at 100 mg/ml concentration) on the oxygen consumption of SGBS adipocytes. (**d**) n = 4 *p < 0.05; **p < 0.01. Expression of mitochondrial creatine kinases (*Ckmt1a* and *Ckmt2*) in SGBS adipocytes. (**e**) Gene expression was determined by RT-qPCR and target genes were normalized to *Gapdh*. n = 3 *p < 0.05.

To test the functional capacity of differentiated SGBS adipocytes, mitochondrial oxygen consumption rate (OCR) was measured (Fig. 3a,b). We found higher basal OCR after the browning was induced by either PPARγ ligand, irisin or BMP7. When we stimulated adipocytes with cAMP, the OCR of browning adipocytes was elevated more robustly compared to the white adipocytes (Fig. 3b). A similar trend was observed as a result of oligomycin treatment which is an inhibitor of H^+^-ATP synthase and provides the measurement of proton leak respiration (Fig. 3b). In parallel, we detected significantly elevated extracellular acidification rate (ECAR) both in untreated and cAMP-stimulated browned adipocytes as compared to white cells (Fig. 3c).

We found it important to determine what proportion of cAMP induced OCR is related to the UCP1 independent creatine phosphate futile cycle, a characteristic feature of beige adipocytes, utilizing ß-guanidinopropionic acid (β-GPA) which is an inhibitor of this cycle^21^. The creatine-cycle related OCR was markedly higher in case of the browned SGBS cells, as well as in irisin and BMP7 treated adipocytes, in contrast to untreated white adipocytes (Fig. 3d). *Ckmt1a* and *Ckmt2* mitochondrial creatine kinases were expressed at a greater extent in the browned SGBS adipocytes as compared to those which were differentiated to white. The expression level of these genes was below the detection limit in undifferentiated preadipocytes (Fig. 3e). This suggests that the creatine kinase futile cycle is active in browned SGBS adipocytes induced either by PPARγ-driven browning differentiation or by the administration of irisin and BMP7 during white differentiation, demonstrating that the browned SGBS adipocytes functionally resemble beige cells.

Notably, *Pm20d1* which encodes the secreted enzyme peptidase M20 domain containing 1 was induced in response to the administration of the browning cocktail and when irisin was added to the white differentiation cocktail (Supplementary Fig. S3). This means that browned SGBS adipocytes have the potential to produce N-acyl amino acids which can function as endogenous uncouplers of UCP1-independent thermogenesis^47^.

### The brown/beige adipocyte phenotype of differentiated SGBS cells is maintained in the absence of PPARγ-ligand

**Figure 4:**
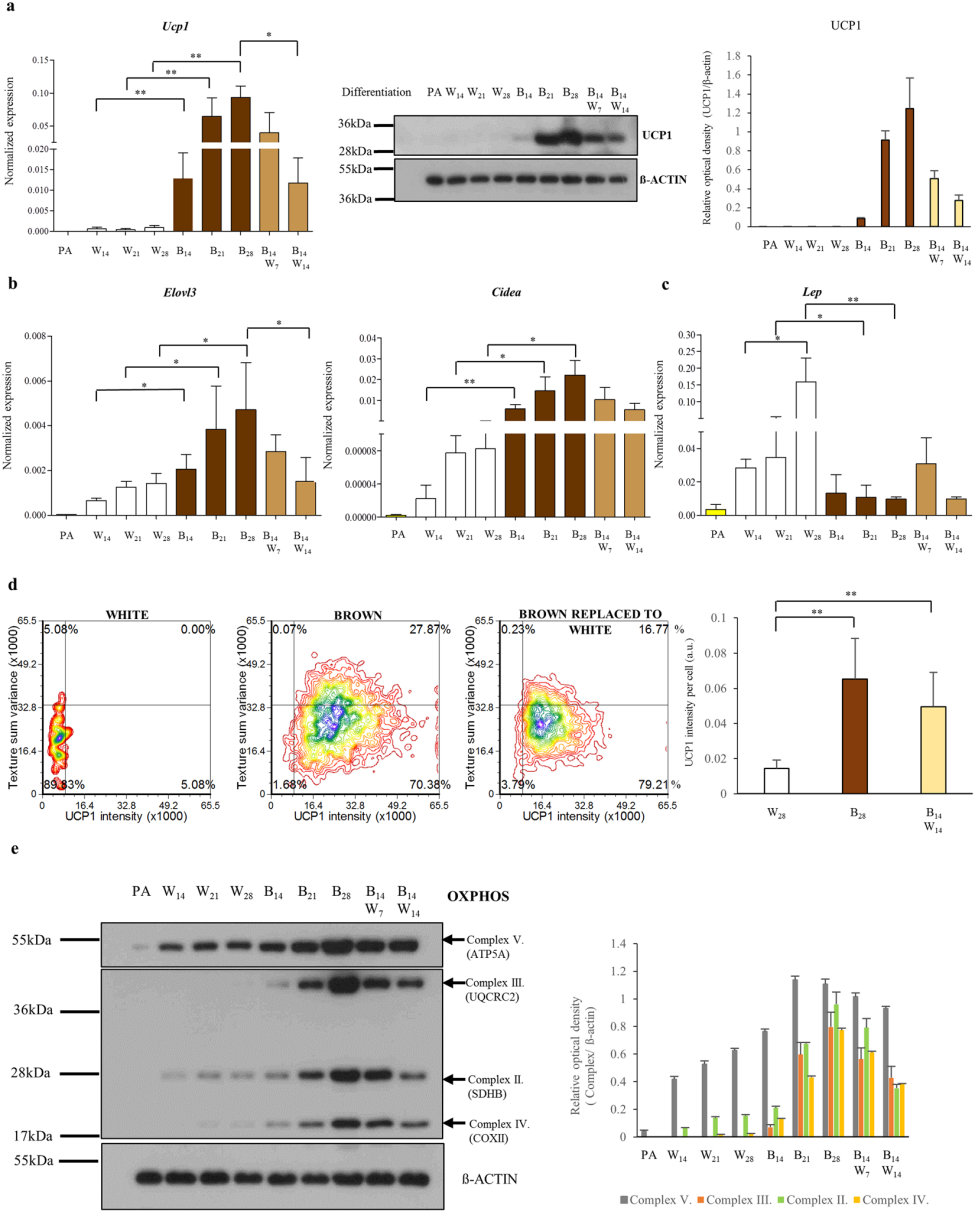
PPARγ-driven differentiation can be maintained in long-term cultures of browning adipocytes. SGBS preadipocytes were differentiated to white (W) or brown (B) for two, three and four weeks (indicated by the number of days), respectively. At day 14, the PPARγ-driven browning cocktail was replaced by the white without rosiglitazone for additional one (B14,W7) or two weeks (B14,W14). Expression of UCP1 at mRNA and protein level **(a)**. Normalized expression of *Cidea*, *Elovl3* **(b)** and *Lep* **(c)** genes by RT-qPCR, n = 4. Density plot figures show texture sum variance and UCP1 protein content of differentiated cells detected by Laser-scanning cytometry in one representative SGBS replicate; n = 3, 1000–2000 cells per each sample **(d)**. Expression of mitochondrial complex subunits detected by immunoblotting **(e)**. Relative optical density was determined by Image J software. ß-actin was used as endogenous control, n = 3. All gels were run under the same conditions. The blots were cropped. The original pictures of the full-length blots are displayed in Supplementary Fig. S7. *p < 0.05; **p < 0.01.

In order to determine whether the PPARγ-driven differentiation could maintain a beige phenotype on a longer time frame, we performed a long-term experiment after 2 weeks for 21 or 28 days; and in parallel samples the browning cocktail was replaced by the white differentiation cocktail for the third (B14,W7) and the fourth week (B14,W14). The highest *Ucp1* mRNA expression was detectable at the end of the long-term browning differentiation program, at day 28 (Fig. 4a). *Ucp1* mRNA level was increased when we replaced the browning cocktail to white, at least until day 21, then sank to the level of day 14. UCP1 protein expression was detected by Western blot at day 14 of PPARγ-driven differentiation and it was further enhanced by after one or two weeks of further stimulation that is long-term rosiglitazone induction resulted in robust upregulation of UCP1 at the protein level. When the PPARγ stimulus was removed after 2 weeks of browning differentiation program, the expression level of UCP1 protein was slightly elevated by the end of the third week, and it was still detectable one week later at a decreased level (Fig. 4a, Supplementary Fig. S7). This was verified with 3 independent anti-UCP1 antibodies (Supplementary Fig. S8).

Parallely, we investigated the expression of *Cidea* and *Elovl3* brown-fat specific genes; similarly to *Ucp1*, further upregulation was observed when we continued the administration of the browning protocol; when PPARγ-stimulation was withdrawn, their expression levels remained elevated as compared to B14 (Fig. 4b). The replacement of the cocktails from browning to white without rosiglitazone did not induce de novo white adipogenesis, marked by the continuous low expression of *Lep* (Fig. 4c).

After four-weeks of differentiation, we observed by immunohistochemistry a six-fold higher UCP1 protein content as a result of the browning cocktail compared to white. When the PPARγ-agonist was replaced by the white differentiation protocol for the last 2 weeks the UCP1 protein content per cell did not change significantly (Fig. 4d) as compared to adipocytes maintained continuously in the presence of the PPARγ-driven differentiation regimen. In parallel, similar texture sum variance was detected in both cell populations (Fig. 4d).

Mitochondrial oxidative phosphorylation (OXPHOS) plays a fundamental role in energy production in brown/beige adipocytes^48^. We found that protein expression of mitochondrial respiratory components in complex II, III and IV followed the pattern of UCP1 expression in SGBS adipocytes (Fig. 4e, Supplementary Fig. S7) that is higher expression of respiratory chain proteins were found in browned adipocytes compared to control white adipocytes indicating stimulated mitochondrial biogenesis. The withdrawal of PPARγ agonist resulted in only a slight reduction of the level these proteins suggesting the clearance of a small fraction of the mitochondria. These results suggest that the signaling pathways driving the beige phenotype in SGBS cells can be maintained at least for two consecutive weeks after the PPARγ agonist is removed.

## Discussion

hADMSCs isolated from SVFs are the most widely used *ex vivo* system to study human adipocyte browning^25–28^. Recently, we showed that hADMSCs from subcutaneous fat could be differentiated into beige adipocytes and we observed upregulation of brown-specific marker proteins (UCP1, CIDEA) in single adipocytes by using laser-scanning cytometry. Furthermore, we demonstrated that irisin could induce beige type of browning when added during white adipocyte differentiation as indicated by *Tbx1* induction while BMP7 addition resulted in *Zic1* induction pointing to the possibility of the classical brown adipocyte differentiation pathway^27^. However, the major limitations of experiments using hADMSCs are the restricted availability of biopsy material and the potentially large variability between donors, which may greatly affect the reproducibility of the obtained data. Fat donors may vary in nutrition, metabolism, life-style and genetic background, e.g. the presence of the risk-allele of the FTO locus (rs1421085), which fundamentally affects adipocyte browning and represents robust genetic association with obesity^31^.

Recently, several attempts were reported to overcome the limitations described above. Artificially immortalized cell lines, like PAZ6 cells, which were obtained from SVF of human infant BAT, and transformed with SV40 T and t antigens were able to differentiate into brown adipocytes *in vitro*^49^. PAZ6 is considered to be a brown preadipocyte cell line, because high UCP1 expression was shown in differentiated PAZ6 cells which could be further induced by norepinephrine^50^. Shinoda *et al*. compared the differentiation of 65 clonal preadipocyte lines originated from supraclavicular fat biopsies with 35 lines which were generated from the SVF of subcutaneous WAT. RNA sequencing was performed from three clonal brown and white adipocyte cultures, differentiated from supraclavicular and subcutaneous progenitor clones, respectively. They found that human browned adipocytes from supraclavicular regions display molecular signatures which resemble thermogenic beige adipocytes^32^. In another study, clonally derived adipocyte progenitors from the deep neck area were capable of differentiating to functional thermogenic adipocytes and responded to BMP7 administration, in contrast to those which were isolated and generated from subcutaneous WAT^33^.

The SGBS human preadipocyte cell line is often used in experiments to study human adipocyte biology and previously it served as a representative model of white adipocyte differentiation^34–37^. The first results, showing the possibility that SGBS cells can differentiate into thermogenic adipocytes were described in 2013. 2-oxoglutarate dependent dioxygenase (encoded by the proposed *Fto* gene) deficient SGBS preadipocytes differentiated to white adipocytes had elevated UCP1 expression and uncoupled respiration, without any changes in mitochondrial mass or structure^39^. Later, Tews *et al*.^40^ found that TENM2 (teneurin-2), which might inhibit the classical brown marker, *Zic1*^51^, is enriched in white adipocyte progenitor cells from subcutaneous neck WAT compared to deep neck ones. They demonstrated that TENM2 knockdown with siRNA in SGBS cells resulted in increased UCP1 at mRNA and protein level. Furthermore, basal and proton leak mitochondrial respiration was enhanced. Interestingly, TENM2 deficient SGBS adipocytes had the same amount of mitochondria as the wild type ones and gained larger lipid droplets than the control cells. The aforementioned results suggest that TENM2 knockdown in SGBS adipocytes results in a beige gene expression program (the expression of *Zic1* did not change) without enhanced mitochondrial biogenesis and accumulation of small lipid droplets in a multilocular arrangement^40^. In these studies, SGBS cells differentiated to white adipocytes for two weeks expressed UCP1 at low, but detectable levels which we could confirm in the present study. In response to the knockdown of 2-oxoglutarate dependent dioxygenase or TENM2 or the administration of the atypical antipsychotic clozapine^28^, the browning potential of the SGBS cells could be significantly enhanced.

In contrast to these studies and to results presented here, Guennoun *et al*. found that the SGBS adipocytes switch their phenotype during a four week-long differentiation program. Within two weeks, they noticed high UCP1 expression and a thermogenic phenotype even as a result of the white differentiation protocol without adding any browning stimuli. Then, the expression of UCP1 greatly declined in response to the continuation of the white differentiation for additional two weeks^52^. Yeo *et al*. seemingly strengthened these results by comparing the gene expression pattern of the differentiated SGBS and primary hADMSC-derived subcutaneous adipocytes^53^. Of note, hADMSCs in this case were obtained from obese patients and there might be a difference between the differentiation capacity of these and healthy hADMSCs and SGBS preadipocytes. They also observed that UCP1 protein expression was high in SGBS cells differentiated for 12 days even when either rosiglitazone or T3 were omitted from the media. In these experiments they considered a band on immunoblots with an apparent molecular mass of approximately 25 kDa as UCP1.

In our experiments, however, we could demonstrate UCP1 protein expression in response to long-term rosiglitazone and T3 administration (browning protocol) at ~33 kDa, but not when the white cocktail was applied. In order to confirm that we take the right band for UCP1, three kinds of anti-UCP1 (both monoclonal and polyclonal) antibodies were used to investigate the UCP1 protein expression. The application of polyclonal antibodies (U6382, PA1-24894) resulted in three distinct bands at ~36 kDa, ~33 kDa and below 28 kDa (Supplementary Fig. S8). However, when a monoclonal antibody (MAB6158) was applied, we could clearly demonstrate which band corresponds to the UCP1 protein, supported by its appearance at the predicted molecular weight of 33 kDa. Furthermore, UCP1 expressed in a mouse BAT lysate was detected at the very same molecular weight as the UCP1 band in the browning SGBS adipocyte samples (Supplementary Fig. S8).

Using laser-scanning cytometry^27^ and applying functional assays we aimed to investigate whether classical brown or beige adipocyte differentiation (browning) can be induced in SGBS cells and if these adipocytes maintain their morphology during long-term culturing. The browning of SGBS cells was induced by multiple approaches: sustained PPARγ stimulation, irisin and BMP7 treatment. Irisin induces browning of subcutaneous white adipocytes *ex vivo*^17,25,27^, BMP7 drives brown fat cell differentiation of both mesenchymal progenitor cells and committed brown preadipocytes. *In vivo*, BMP7 is able to increase brown fat mass and thermogenic energy expenditure in mice^18^. The present study demonstrates that the PPARγ-driven browning protocol (including rosiglitazone) and irisin treatment could be successfully used to induce browning of SGBS adipocytes, which resulted in a beige phenotype. BMP7 administration had a more moderate effect and induced a distinct gene expression program without the upregulation of the beige-selective markers, *Tbx1* and *Cited1*. Both in primary subcutaneous and in SGBS adipocytes, BMP7 treatment enhanced the expression of the *Pparg* and *Zic1* genes suggesting that this mediator rather induce a classical brown-like differentiation^27^. This program, similarly to irisin administration, resulted in increased mitochondrial DNA and *Ucp1* levels and elevated mitochondrial respiration. Of note, the majority of the browning marker genes were detectable in white SGBS adipocytes but their expression remained markedly lower compared to the browned cells.

The functional experiments detected UCP1-dependent proton leak (heat production) and high extracellular acidification in the case of beige adipocytes induced by either rosiglitazone or irisin. These parameters were significantly lower in the case of white adipocytes, however, could be stimulated in response to cAMP supporting the notion that some of the differentiated white adipocytes are masked beige cells. Besides UCP1-dependent thermogenesis, UCP1-independent heat-producing mechanisms were described as a beige specific feature. In our experiments, the involvement of the creatine-phosphate futile cycle^21^ was investigated in the heat production of SGBS beige adipocytes. Using a specific compound (β-GPA) which depletes creatine and inhibits the cycle, we demonstrated the induction of this futile cycle in response to a β-adrenergic cue in beige SGBS adipocytes.

Several single nucleotide polymorphisms located in the fat mass and obesity-associated gene, *Fto* were described by genome-wide association studies as a genetic trait predisposing for obesity^54,55^. The T-to-C conversion at rs1421085 position affects a mesenchymal super-enhancer site and leads to increased IRX3 and IRX5 expression levels during early adipocyte differentiation, because of the lack of ARID5B binding and repression, which results in a developmental shift from energy-dissipating beige cells to energy-storing white adipocytes. Therefore, the potential of mitochondrial thermogenesis is reduced in those individuals who carry the C risk allele which occurs at a high frequency (approx. 44%) in the European population^31^. So far no information has been available about the status of this allele in SGBS cells. A CC to TT rescue by genome editing of primary adipocytes resulted in an elevated *Ucp1* expression by two and an increased stimulated oxygen consumption by seven fold^31^. To our knowledge, no data is available how a heterozygous composition affects these parameters. In this study, we demonstrated that the SGBS cell line carries one copy of the C risk allele; still these preadipocytes were able to differentiate into functional beige adipocytes if browning stimuli (e.g. rosiglitazone or irisin treatment) were continuously present during their differentiation.

In summary, the induction of browning in SGBS cells followed the beige pathway, as a result of the PPARγ-driven differentiation (rosiglitazone) or irisin treatment. The beige phenomenon is supported by the elevated expression of brown and beige marker genes, multilocular morphology, enrichment of mitochondria, high oxygen consumption in response to cAMP and significant involvement of the creatine-phosphate futile cycle in heat production. In our experiments, when the browning-inducer, rosiglitazone was eliminated from the differentiation media, UCP1 expression and mitochondrial enrichment could be partially maintained. In summary, we propose that the human SGBS preadipocyte cells represent an uncommitted preadipocyte stage with one FTO risk allele, which are able to differentiate into both white and beige adipocytes and can be used as an easily applicable and valuable model to study human adipocyte browning in a reproducible as well as high-throughput manner contributing to the development of novel therapeutic approaches against obesity.

## Materials and Methods

### Cell culture- and differentiation of human SGBS preadipocytes into mature white and brown adipocytes

SGBS preadipocytes were seeded in 12-well plates (Costar) and cultured in DMEM-F12 (Dulbecco’s Modified Eagle’s Medium/Nutrient F-12 Ham) medium (Sigma, Germany) containing 33 µM biotin (Sigma), 17 µM pantothenic acid (Sigma), 100U/ml penicillin/streptomycin (Sigma) and 10% FBS (Gibco, UK) at 37 °C in 5% CO_2_ for overnight to attach. Cells were cultured in this medium until they reach complete confluency. The absence of mycoplasma was checked by polymerase chain reaction (PCR) analysis (PCR Mycoplasma Test Kit I/C, PromoKine, PromoCell France). White adipogenic differentiation was induced for four days using serum-free medium supplemented with 33 µM biotin, 17 µM pantothenic acid, 100U/ml penicillin/streptomycin, 2 µM rosiglitazone (Cayman Chemicals, USA), 10 µg/ml human apo-transferrin (Sigma), 20 nM human insulin (Sigma), 25 nM dexamethasone (Sigma), 500 µM 3-isobutyl-1-methylxantine (Sigma), 100 nM cortisol (Sigma) and 200pM triiodothyronine (Sigma). After the fourth day, the medium was changed; and rosiglitazone, 3-isobutyl-1-methylxantine and dexamethasone were removed during the remaining 10 days of differentiation. The differentiation medium was replaced in every fourth day^36^.

PPARγ-driven browning differentiation was induced for four days using serum-free medium supplemented with 33 µM biotin, 17 µM pantothenic acid, 100U/ml penicillin/streptomycin, 10 µg/ml human apo-transferrin, 0,85 mM human insulin, 1 µM dexamethasone, 500 µM 3-isobutyl-1-methylxantine and 200pM triiodothyronine. After four days, the medium was changed for following ten days adding 500 nM rosiglitazone while dexamethasone and 3-isobutyl-1-methylxantine were omitted^29^. In long-term experiments either the same culture conditions were applied for 21 and 28 days or it was replaced by the white differentiation medium without rosiglitazone, 3-isobutyl-1-methylxantine and dexamethasone. The differentiation medium was changed in every fourth day.

Human recombinant irisin (Cayman Chemicals) was used at 250 ng/ml concentration, and human recombinant BMP7 (R&D systems, Minneapolis, USA) was administered at 50 ng/ml concentration^26,27^. Where indicated, the browning-inducers were added to the differentiating cells in increasing concentrations.

### RNA isolation, RT-PCR, qPCR

Total RNA was isolated from differentiated SGBS adipocytes by using Trizol reagent (Invitrogen Life Technologies, USA). RNA concentrations were determined by spectrophotometry. To generate cDNA, TaqMan reverse transcription reagents kit (Applied Biosystems, USA) was applied according to the manufacturer’s instructions. Applied Biosystems designed the gene primers and probes. Quantitative real-time PCR (qPCR) was performed in a LightCycler 480 (Roche) with the program of 10 seconds at 94 °C, followed by 40 cycles of 12 seconds at 94 °C and 30 seconds at 60 °C. All samples were used in triplicates. Normalized gene expressions were calculated by ∆Ct method. Human *Gapdh* was used as an endogenous control^27^.

### Mitochondrial DNA isolation and quantification by qPCR

Total DNA was isolated from differentiated SGBS adipocytes by the phenol-chloroform extraction, using Trizol reagent (Invitrogen Life Technologies). During the qPCR we were using diluted samples, 10 µM from each primer (human mitochondrial DNA specific primers: forward 5′CTATGTCGCAGTATCTGTCTTTG-3′, reverse 5′-GTTATGATGTCTGTGTGGAAAG-3′, nuclear specific primers (SIRT1 gene): forward 5′CTTTGTGTGCTATAGATGATATGGTAAATTG-3′, reverse 5′GATTAAACAGTGTACAAAAGTAG-3′), and Maxima SYBR Green/ROX qPCR Master Mix (Thermo Scientific, USA). LightCycler 480 (Roche) was used with the program of 20 minutes at 95 °C, and 50 cycles of 15 seconds at 95 °C, 20 seconds at 58 °C, 20 seconds at 72 °C. Relative mitochondrial DNA content was calculated from the difference between the threshold cycle (CT) values for mitochondrial DNA and nuclear specific amplification. Data show mitochondrial genomes per diploid nuclei^27,28,56^.

### Antibodies and immunoblotting

Differentiated SGBS adipocytes and undifferentiated control cells were washed once with PBS, and then suspended in lysis buffer which consists of 50 nM Tris-HCl, 0,1% Triton X-100 (Sigma), 15 mM 2-mercaptoethanol (Sigma), 1 mM EDTA (Sigma) and protease inhibitor (Sigma). The lysates were suspended in 5x Laemmli loading buffer and boiled for 10 min at 100 °C. Equal amount of proteins were loaded onto a 12%-SDS-polyacrylamide gel, and transferred onto a PVDF Immobilon-P Transfer Membrane (Merck-Millipore, Germany). Then the membranes were blocked in 5% skimmed milk (Sigma) for 1 hour. For overnight, membranes were probed at 4 °C with primary antibodies: monoclonal anti-UCP1 (1:1000, R&D Systems, MAB6158), polyclonal anti-UCP1 (1:500, Sigma, U6382; 1:500, Thermo Scientific, PA1-24894), anti-OXPHOS (1:1000, Abcam, UK, ab110411), and anti-actin (1:10000, Sigma, A2066), in TBS-T containing 1% non-fat skimmed milk, followed by the incubation with horseradish-peroxidase-conjugated species corresponding secondary antibodies (Sigma) for 1 hour at room temperature. Immunoreactive proteins were visualized by Immobilion western chemiluminescence substrate (Merck-Millipore). ImageJ software was used to carry out the densitometry measurements.

### Oxygen consumption and extracellular acidification measurements

Real-time oxygen consumption and extracellular acidification rates were measured by using an XF96 oximeter (Seahorse Biosciences, North Billerica, MA, USA). SGBS cells were seeded onto 96-well XF96 cell culture microplates. Cells were kept in growth medium at the longest for 24 hours and then the formerly-described differentiation process started. After recording the baseline oxygen consumption, cells received a single bolus of dibutyril-cAMP at 500 µM concentration to induce adrenergic stimulation. Then, stimulated oxygen consumption was measured in every 30 minutes. The final reading took place at 6 h post-treatment. Differentiated adipocytes were treated with 2 mM ß-guanidinopropionic acid (Sigma) to block the creatine-driven substrate cycle^21^. In addition, proton leak respiration was measured by oligomycin (Enzo, USA) treatment at 2 µM concentration, which blocks the ATP synthase. For baseline correction, cells received a single bolus of Antimycin A (Sigma) treatment at 10 µM concentration. After the measurements, oxygen consumption rate was normalized to protein content^27,28,56^.

### Laser-scanning cytometry

SGBS cells were plated on eight-well ibidi micro slides (Ibidi GmbH, Germany) and differentiated as previously described. Immunofluorescence staining was carried out as described previously^27^. Scanning was done by an iCys Research Imaging Cytometer (iCys, Thorlabs Imaging Systems, Sterling, VA, USA). The images were processed and analyzed (n = 3, 2000 cells per SGBS sample) by our high-throughput automatic cell-recognition protocol^27^ with some modifications using the iCys companion software (iNovator Application Development Toolkit version 7.0, CompuCyte Corporation, Westwood, MA, USA). As previuosly, cells were identified according to their Hoeschst 33342 nuclear staining. Then, the fluorescence signal intensity of the UCP1 immunostaining and the texture sum variance of the light scatter signal of lipid droplets were quantified in each cell within the 30-pixel immediate outward vicinity of the nucleus contour by the Cell Profiler software (The Broad Institute of MIT, MA, USA). Afterward, based on these fluorescence and light scatter signal of single cells, a semi-automated classification and enumeration of the differentiated white and brown adipocytes and undifferentiated preadipocytes was carried out applying the trained classification “Fast Gentle Boosting” of the Cell Profiler Analyst software (The Broad Institute of MIT, MA, USA).

### Flow cytometry

To investigate the phenotype of the undifferentiated SGBS preadipocytes, a multiparametric analysis of surface antigen expression was performed by three-color flow cytometry using fluorochrome-conjugated antibodies with isotype matching controls. See reference ref.^41^ for further details about the analysis^41^.

### Genotyping

Genomic DNA was purified with GeneJET Genomic DNA Purification Kit (Thermo Scientific) according to the manufacturer’s protocol. Rs1421085 SNP (single nucleotide polymorphism) was genotyped by qPCR using TaqMan genotyping assays and by DNA sequencing. To amplify the corresponding genomic region, we designed the following primer pair: Forward: 5′GATGACACACACCATGAGCC, Reverse: 5′TAACAGTGGAGGTCAGCACA. Following PCR amplification, we purified the PCR product with NucleoSpin® Gel and PCR Clean-up kit (Macherey-Nagel, Germany). Then the quality of the product was investigated by 2% agarose gel electrophoresis. DNA was sequenced by Sanger sequencing method.

### Statistical analysis

Results are expressed as the mean ± SD for the number of assays indicated. For multiple comparisons of groups statistical significance was calculated and evaluated by one-way ANOVA followed by Tukey post-hoc test. To compare two groups, two-tailed paired Student’s t-test was used.

## Supplementary information

Supplementary Information

## Data Availability

All data generated and analyzed during this study are included in this manuscript (and its Supplementary Information files).

## References

[1] Lynes MD, Tseng Y-H (2015). The thermogenic circuit: regulators of thermogenic competency and differentiation. Genes & diseases.

[2] Ishibashi J, Seale P (2010). Beige can be slimming. Science.

[3] Petrovic N (2010). Chronic peroxisome proliferator-activated receptor γ (PPARγ) activation of epididymally derived white adipocyte cultures reveals a population of thermogenically competent, UCP1-containing adipocytes molecularly distinct from classic brown adipocytes. Journal of Biological Chemistry.

[4] Cousin B (1992). Occurrence of brown adipocytes in rat white adipose tissue: molecular and morphological characterization. Journal of cell science.

[5] Collins S, Daniel KW, Petro AE, Surwit RS (1997). Strain-Specific Response toβ 3-Adrenergic Receptor Agonist Treatment of Diet-Induced Obesity in Mice. Endocrinology.

[6] Ohno H, Shinoda K, Spiegelman BM, Kajimura S (2012). PPARγ agonists induce a white-to-brown fat conversion through stabilization of PRDM16 protein. Cell metabolism.

[7] Lidell ME (2013). Evidence for two types of brown adipose tissue in humans. Nature medicine.

[8] Fedorenko A, Lishko PV, Kirichok Y (2012). Mechanism of fatty-acid-dependent UCP1 uncoupling in brown fat mitochondria. Cell.

[9] Rosen ED, Spiegelman BM (2014). What we talk about when we talk about fat. Cell.

[10] Timmons JA (2007). Myogenic gene expression signature establishes that brown and white adipocytes originate from distinct cell lineages. Proc. Natl. Acad. Sci. USA.

[11] Long JZ (2014). A smooth muscle-like origin for beige adipocytes. Cell metabolism.

[12] Sharp LZ (2012). Human BAT possesses molecular signatures that resemble beige/brite cells. PLoS One.

[13] Kajimura S, Spiegelman BM, Seale P (2015). Brown and Beige Fat: Physiological Roles beyond Heat Generation. Cell metabolism.

[14] Waldén TB, Hansen IR, Timmons JA, Cannon B, Nedergaard J (2011). Recruited vs. nonrecruited molecular signatures of brown,“brite,” and white adipose tissues. American Journal of Physiology-Endocrinology and Metabolism.

[15] Kim H (2018). Irisin Mediates Effects on Bone and Fat via alphaV Integrin Receptors. Cell.

[16] Zhang Y (2014). Irisin stimulates browning of white adipocytes through mitogen-activated protein kinase p38 MAP kinase and ERK MAP kinase signaling. Diabetes.

[17] Boström P (2012). A PGC1-α-dependent myokine that drives brown-fat-like development of white fat and thermogenesis. Nature.

[18] Tseng Y-H (2008). New role of bone morphogenetic protein 7 in brown adipogenesis and energy expenditure. Nature.

[19] Schulz TJ (2013). Brown-fat paucity due to impaired BMP signalling induces compensatory browning of white fat. Nature.

[20] Bordicchia M (2012). Cardiac natriuretic peptides act via p38 MAPK to induce the brown fat thermogenic program in mouse and human adipocytes. J Clin Invest.

[21] Kazak L (2015). A creatine-driven substrate cycle enhances energy expenditure and thermogenesis in beige fat. Cell.

[22] Kazak L (2017). Genetic Depletion of Adipocyte Creatine Metabolism Inhibits Diet-Induced Thermogenesis and Drives Obesity. Cell metabolism.

[23] Cypess AM (2013). Anatomical localization, gene expression profiling and functional characterization of adult human neck brown fat. Nature medicine.

[24] Jespersen NZ (2013). A classical brown adipose tissue mRNA signature partly overlaps with brite in the supraclavicular region of adult humans. Cell metabolism.

[25] Lee P (2014). Irisin and FGF21 are cold-induced endocrine activators of brown fat function in humans. Cell metabolism.

[26] Raschke S (2013). Evidence against a beneficial effect of irisin in humans. PLoS One.

[27] Kristóf E, Doan-Xuan Q-M, Bai P, Bacso Z, Fésüs L (2015). Laser-scanning cytometry can quantify human adipocyte browning and proves effectiveness of irisin. Scientific reports.

[28] Kristof E (2016). Clozapine modifies the differentiation program of human adipocytes inducing browning. Transl Psychiatry.

[29] Elabd C (2009). Human Multipotent Adipose‐Derived Stem Cells Differentiate into Functional Brown Adipocytes. Stem cells.

[30] Silva FJ (2014). Metabolically active human brown adipose tissue derived stem cells. Stem Cells.

[31] Claussnitzer M (2015). FTO obesity variant circuitry and adipocyte browning in humans. New England Journal of Medicine.

[32] Shinoda K (2015). Genetic and functional characterization of clonally derived adult human brown adipocytes. Nature medicine.

[33] Xue R (2015). Clonal analyses and gene profiling identify genetic biomarkers of the thermogenic potential of human brown and white preadipocytes. Nature medicine.

[34] Wabitsch M (2001). Characterization of a human preadipocyte cell strain with high capacity for adipose differentiation. International journal of obesity.

[35] Allott EH (2012). The SGBS cell strain as a model for the *in vitro* study of obesity and cancer. Clinical and Translational Oncology.

[36] Fischer-Posovszky P, Newell FS, Wabitsch M, Tornqvist HE (2008). Human SGBS cells–a unique tool for studies of human fat cell biology. Obesity facts.

[37] Schlottmann I, Ehrhart-Bornstein M, Wabitsch M, Bornstein S, Lamounier-Zepter V (2014). Calcium-dependent release of adipocyte fatty acid binding protein from human adipocytes. International journal of obesity.

[38] Palominos MM, Dünner NH, Wabitsch M, Rojas CV (2015). Angiotensin II directly impairs adipogenic differentiation of human preadipose cells. Molecular and cellular biochemistry.

[39] Tews D (2013). FTO deficiency induces UCP-1 expression and mitochondrial uncoupling in adipocytes. Endocrinology.

[40] Tews D (2017). Teneurin-2 (TENM2) deficiency induces UCP1 expression in differentiating human fat cells. Molecular and cellular endocrinology.

[41] Sárvári AK, Veréb Z, Uray IP, Fésüs L, Balajthy Z (2014). Atypical antipsychotics induce both proinflammatory and adipogenic gene expression in human adipocytes *in vitro*. Biochemical and biophysical research communications.

[42] Wu J (2012). Beige adipocytes are a distinct type of thermogenic fat cell in mouse and human. Cell.

[43] Perdikari A (2018). BATLAS: Deconvoluting Brown Adipose Tissue. Cell Rep.

[44] Cheng Y (2018). Prediction of Adipose Browning Capacity by Systematic Integration of Transcriptional Profiles. Cell Rep.

[45] Jedrychowski MP (2015). Detection and quantitation of circulating human irisin by tandem mass spectrometry. Cell metabolism.

[46] Abdul-Rahman O (2016). AMP-Activated Kinase (AMPK) Activation by AICAR in Human White Adipocytes Derived from Pericardial White Adipose Tissue Stem Cells Induces a Partial Beige-Like Phenotype. PLoS One.

[47] Long JZ (2016). The Secreted Enzyme PM20D1 Regulates Lipidated Amino Acid Uncouplers of Mitochondria. Cell.

[48] Hüttemann M, Lee I, Samavati L, Yu H, Doan JW (2007). Regulation of mitochondrial oxidative phosphorylation through cell signaling. Biochimica et Biophysica Acta (BBA) - Molecular Cell Research.

[49] Zilberfarb V (1997). Human immortalized brown adipocytes express functional beta3-adrenoceptor coupled to lipolysis. Journal of Cell Science.

[50] Kazantzis M (2012). PAZ6 cells constitute a representative model for human brown pre-adipocytes. Frontiers in endocrinology.

[51] Bagutti C, Forro G, Ferralli J, Rubin B, Chiquet-Ehrismann R (2003). The intracellular domain of teneurin-2 has a nuclear function and represses zic-1-mediated transcription. Journal of cell science.

[52] Guennoun A (2015). Comprehensive molecular characterization of human adipocytes reveals a transient brown phenotype. Journal of translational medicine.

[53] Yeo CR (2017). SGBS cells as a model of human adipocyte browning: a comprehensive comparative study with primary human white subcutaneous adipocytes. Scientific reports.

[54] Frayling TM (2007). A common variant in the FTO gene is associated with body mass index and predisposes to childhood and adult obesity. Science.

[55] Scuteri A (2007). Genome-wide association scan shows genetic variants in the FTO gene are associated with obesity-related traits. PLoS genetics.

[56] Szántó M (2011). Poly (ADP-ribose) polymerase-2 depletion reduces doxorubicin-induced damage through SIRT1 induction. Cardiovascular research.

