# Supplementary material for: Differentiating SGBS adipocytes respond to PPARγ stimulation, irisin and BMP7 by functional browning and beige characteristics
Source: Sci Rep. 2019 Apr 9;9:5823. doi: 10.1038/s41598-019-42256-0 (PMC6456729; doi:10.1038/s41598-019-42256-0)
Supplement: Supplementary file 1 — Supplementary Information [file 41598_2019_42256_MOESM1_ESM.pdf]

Supplementary information

Differentiating SGBS adipocytes respond to PPARγ stimulation, irisin and BMP7 by functional browning and beige characteristics

Ágnes Klusóczki, Zoltán Veréb, Attila Vámos, Pamela Fischer Posovszky, Martin Wabitsch, Zsolt Bacsó, László Fésüs*, Endre Kristóf*

a

		Percentage of positive cells (SGBS)	Percentage of positive cells (adipocyte-derived stem cells) ref.41
Hematopoietic/ Monocyte markers	CD34	91.50	3.23
	CD45	0.00	0.00
	CD47	94.44	99.31
	CD338 (ABCG2)	11.81	not determined
	HLA-DR	87.25	3.73
Endothelial markers	CD31 (PECAM)	0.00	6.69
	CD54 (ICAM-1)	74.64	not determined
MSC/Fibroblast markers	CD73	98.73	98.37
	CD90 (Thy-1)	98.74	95.05
	CD105 (Endoglin)	80.32	93.20
Integrins and CAMs	CD18	0.00	not determined
	CD29 (Integrin ß1)	98.40	99.81
	CD36	80.76	not determined
	CD44 (H-CAM,Hermes)	94.92	78.79
	CD49a (Integrin α1)	74.39	99.52
	CD49d (Integrin α4)	0.00	not determined
	CD146 (MCAM)	85.70	65.27
	CD325	96.36	not determined

b

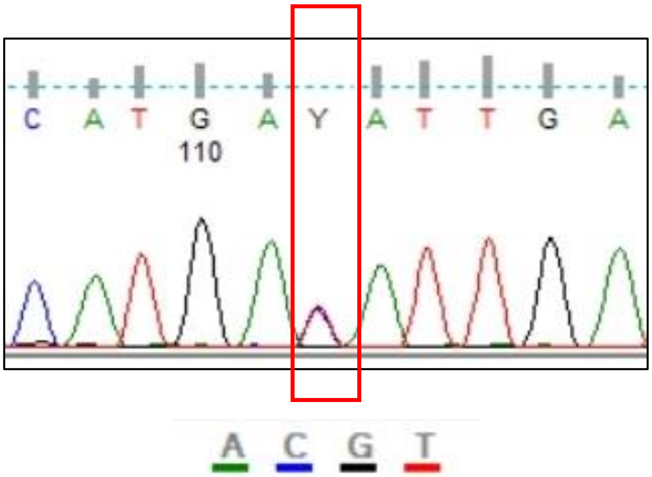

Supplementary Figure S1. Analysis of the surface antigen pattern and the rs1421085 SNP of SGBS preadipocytes. Expression of 18 markers was determined in undifferentiated SGBS preadipocytes by flow cytometry. Four groups of markers were tested: hematopoietic/monocyte, endothelial, MSC/fibroblast markers, integrins and CAMs. The numbers represent the percentage of positive cells **(a)**. Sequence of the rs1421085 locus in the *Fto* gene showing that SGBS cells are heterozygous for the presence of the C risk allele. DNA was sequenced by the Sanger sequencing method **(b)**.

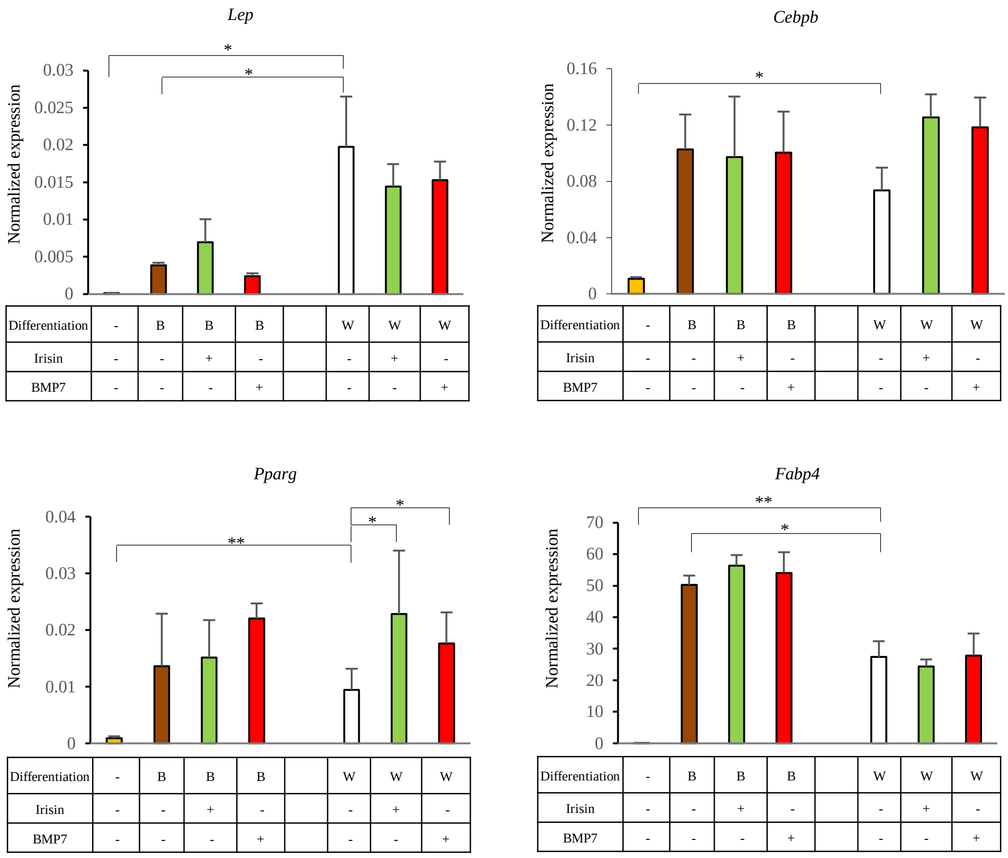

Supplementary Figure S2. Expression of adipogenic markers (*Lep, Cebpb, Pparg, Fabp4*) in SGBS adipocytes. SGBS preadipocytes were differentiated to white (W) or beige (B) for two weeks; human recombinant Irisin treatment at 250 ng/ml concentration (green bars) or BMP7 treatment at 50 ng/ml concentration (red bars) were applied to induce browning of SGBS cells for 14 days. Gene expression was determined by RT-qPCR and target genes were normalized to *Gapdh*. n=5 *p<0.05; **p<0.01

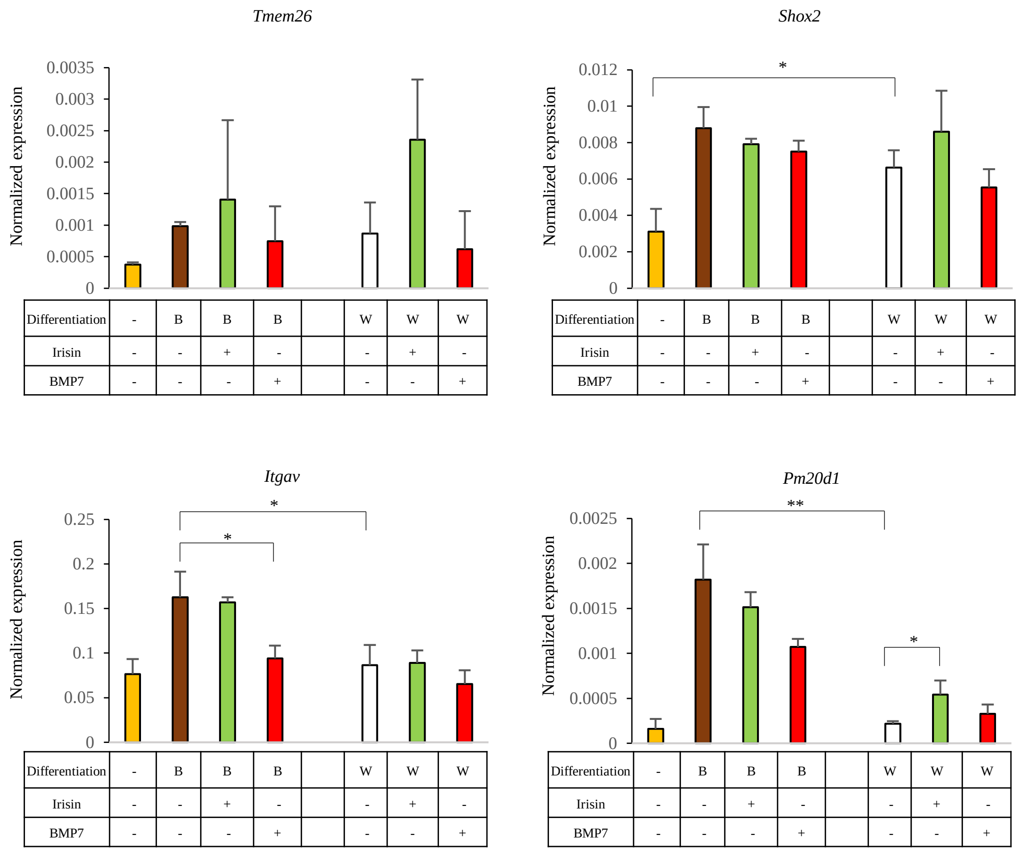

Supplementary Figure S3. Expression of proposed beige markers (*Tmem26, Shox2, Pm20d1*) and the subunit of irisin receptor (*Itgav*) in SGBS adipocytes. SGBS preadipocytes were differentiated to white (W) or beige (B) for two weeks; human recombinant Irisin treatment at 250 ng/ml concentration (green bars) or BMP7 treatment at 50 ng/ml concentration (red bars) were applied to induce browning of SGBS cells for 14 days. Gene expression was determined by RT-qPCR and target genes were normalized to *Gapdh*. n=3 *p<0.05; **p<0.01

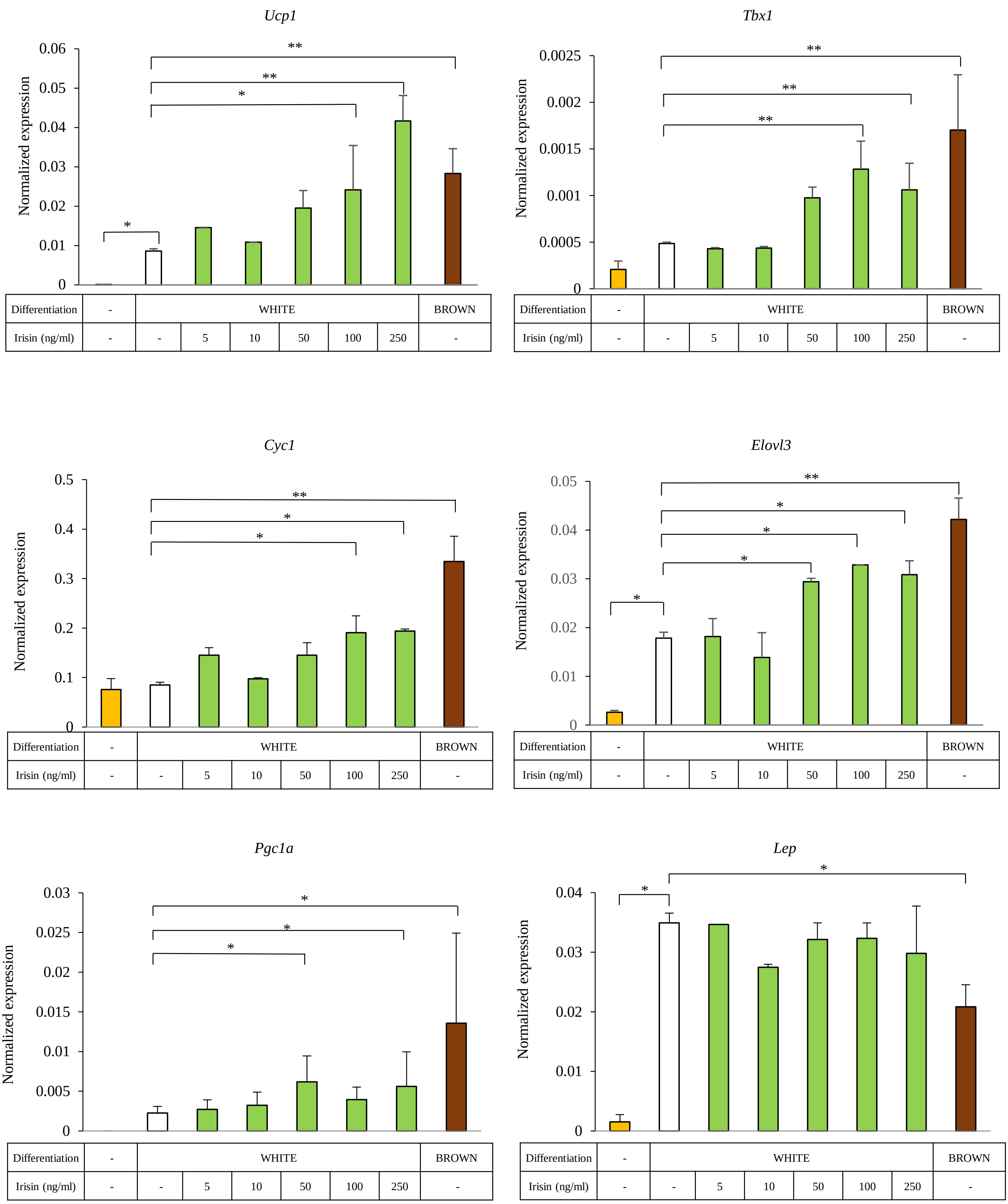

Supplementary Figure S4. Concentration dependence of the Irisin effect during the white adipocyte differentiation program of SGBS cells. Expression of *Ucp1*, *Tbx1*, *Cyc1*, *Elovl3*, *Pgc1a* and *Lep* genes in SGBS adipocytes which were harvested after 14 days differentiation. Gene expression was determined by RT-qPCR and genes were normalized to *Gapdh*. *p<0.05; **p<0,01, n=3

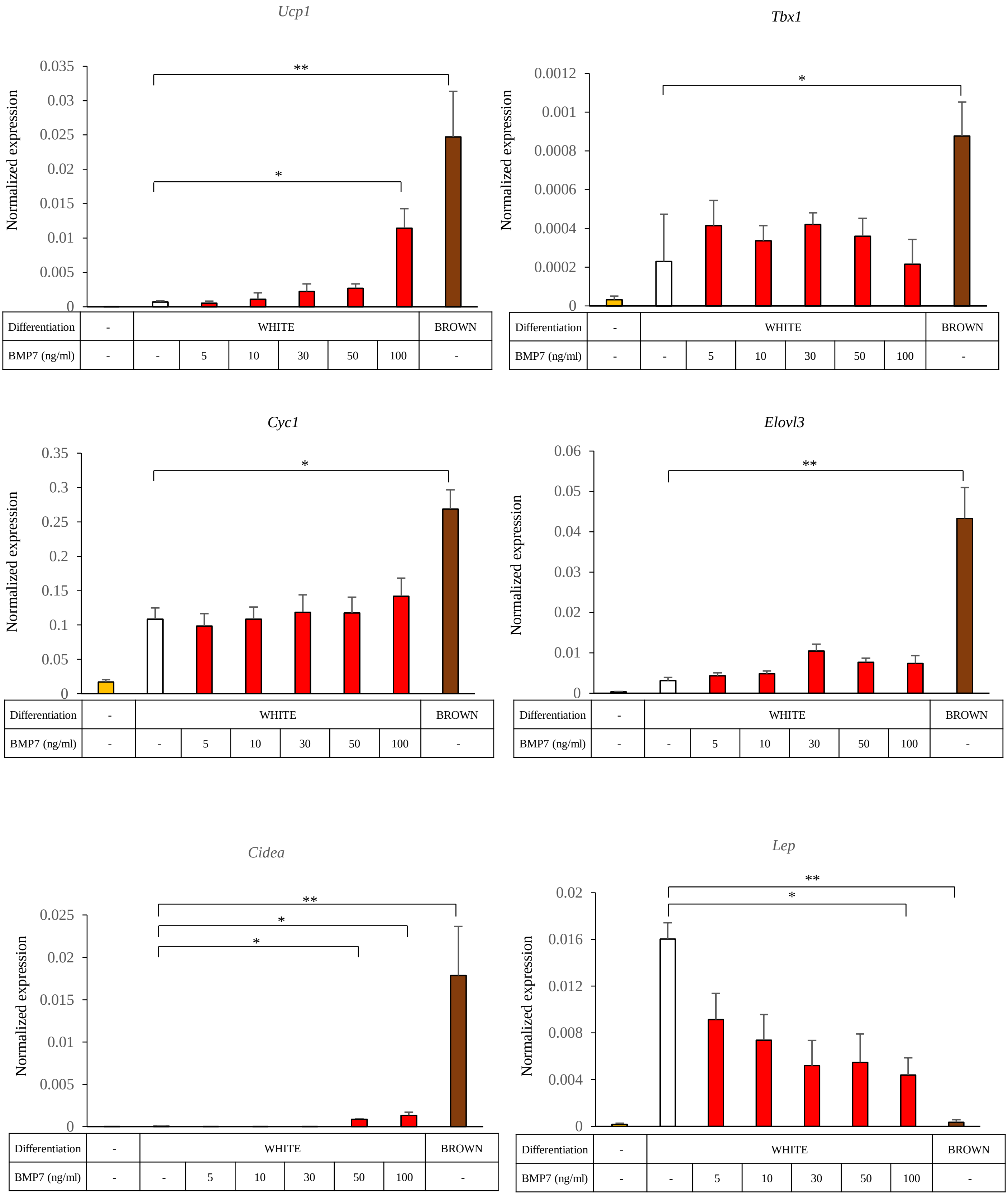

Supplementary Figure S5. Concentration dependence of the BMP7 effect during the white adipocyte differentiation program of SGBS cells. Expression *of Ucp1*, *Tbx1*, *Cyc1*, *Elovl3*, *Cidea* and *Lep* genes in SGBS adipocytes which were harvested after 14 days differentiation. Gene expression was determined by RT-qPCR and genes were normalized to *Gapdh*. *p<0.05; **p<0,01, n=3

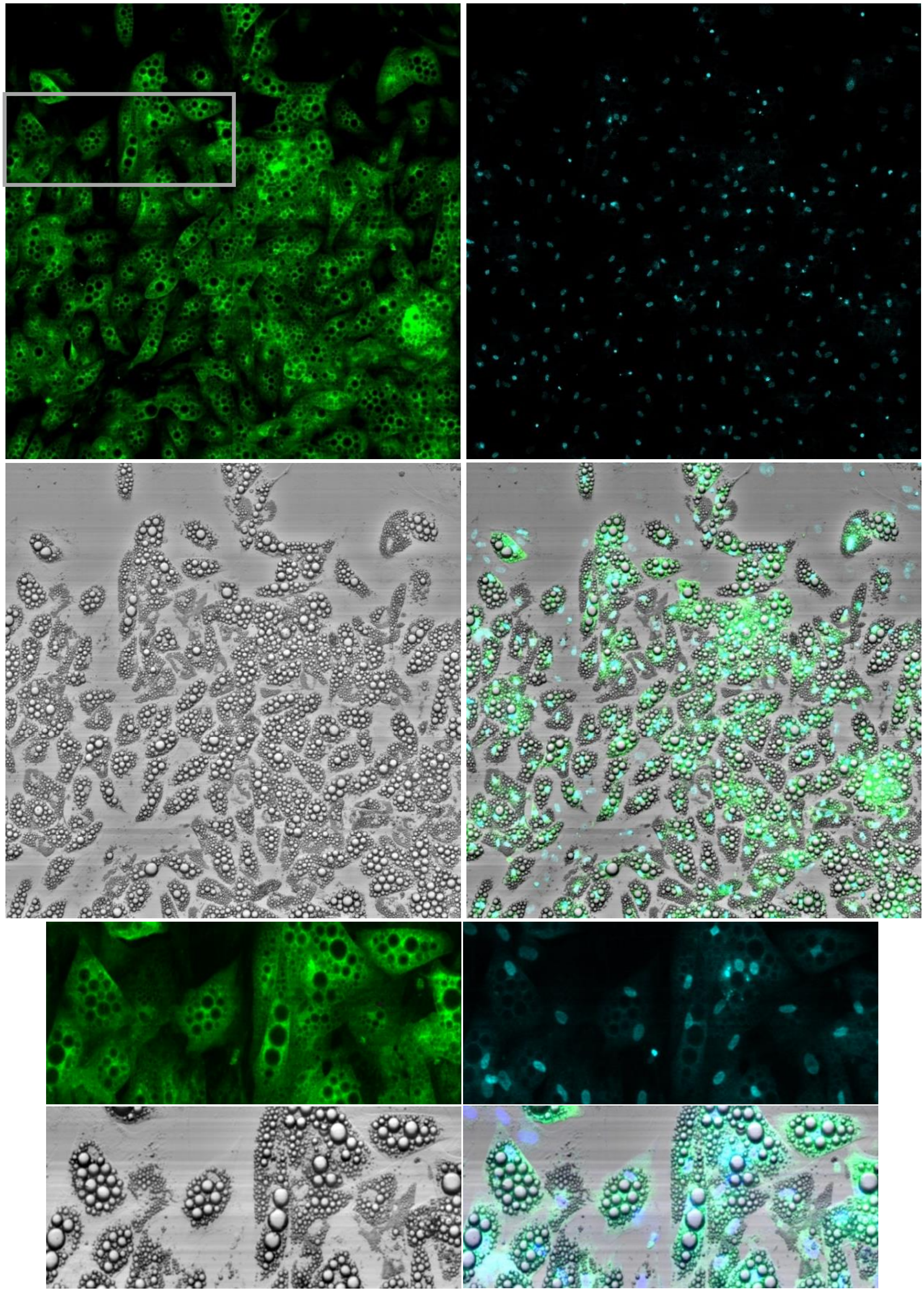

Supplementary Figure S6. Well images (size: width = 996 μm, height = 957 μm, four images in the upper panel) **and field images** (four images in the bottom panel) **of the green** (UCP1 – Alexa-488 labeled indirect immunostaining, upper left image in both panels)**, blue** (nuclei - Hoechst 33342 staining, upper right images) **and light scatter** (bottom left images) **channels and merged images of the previous three channels** (bottom right images) **captured by the iCys laser-scanning cytometer.** This well image, which is an overview image of an appropriately selected area of the sample is composed of 2 x 5 higher resolution field images in the three channels. One of these higher resolution field images in each channel is shown in the bottom panel. The original location of this filed image is indicated by the grey rectangular line in the top left UCP1 channel well image. In every imaging measurements, approximately 5-20 well images were manually selected and automatically recorded. In the image analysis step, individual cells were identified and classified and in the Fig.2A mosaic image 9 of these single cells are shown.

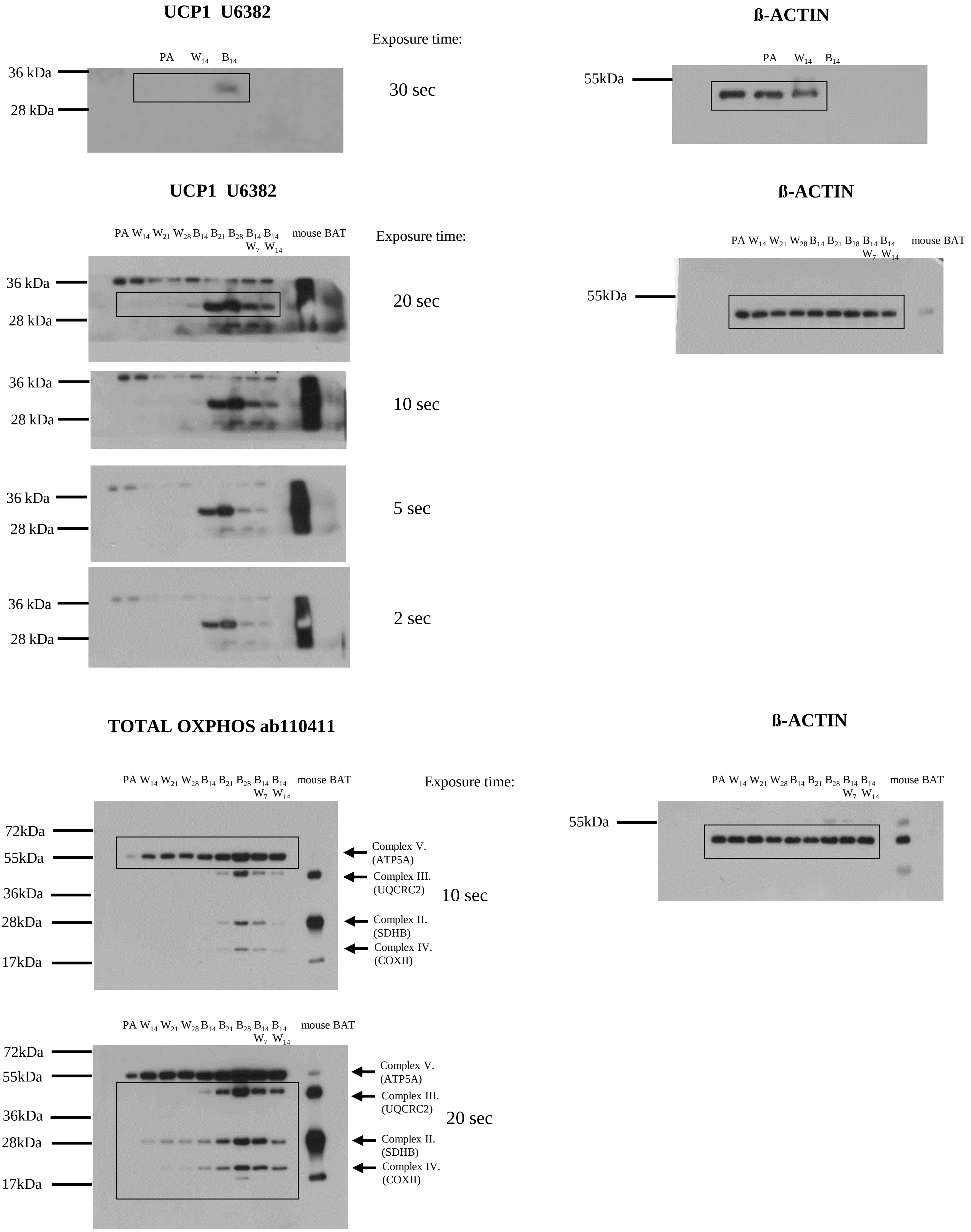

Supplementary Figure S7. Uncropped images presented with molecular weight ladders and a positive control, using U6382 polyclonal anti-UCP1 antibody or anti-OXPHOS antibody as shown in Figure 2.C, Figure 4.A or Figure 4.E. ß-actin was used as endogenous control. Cropped areas are shown in black box regions.

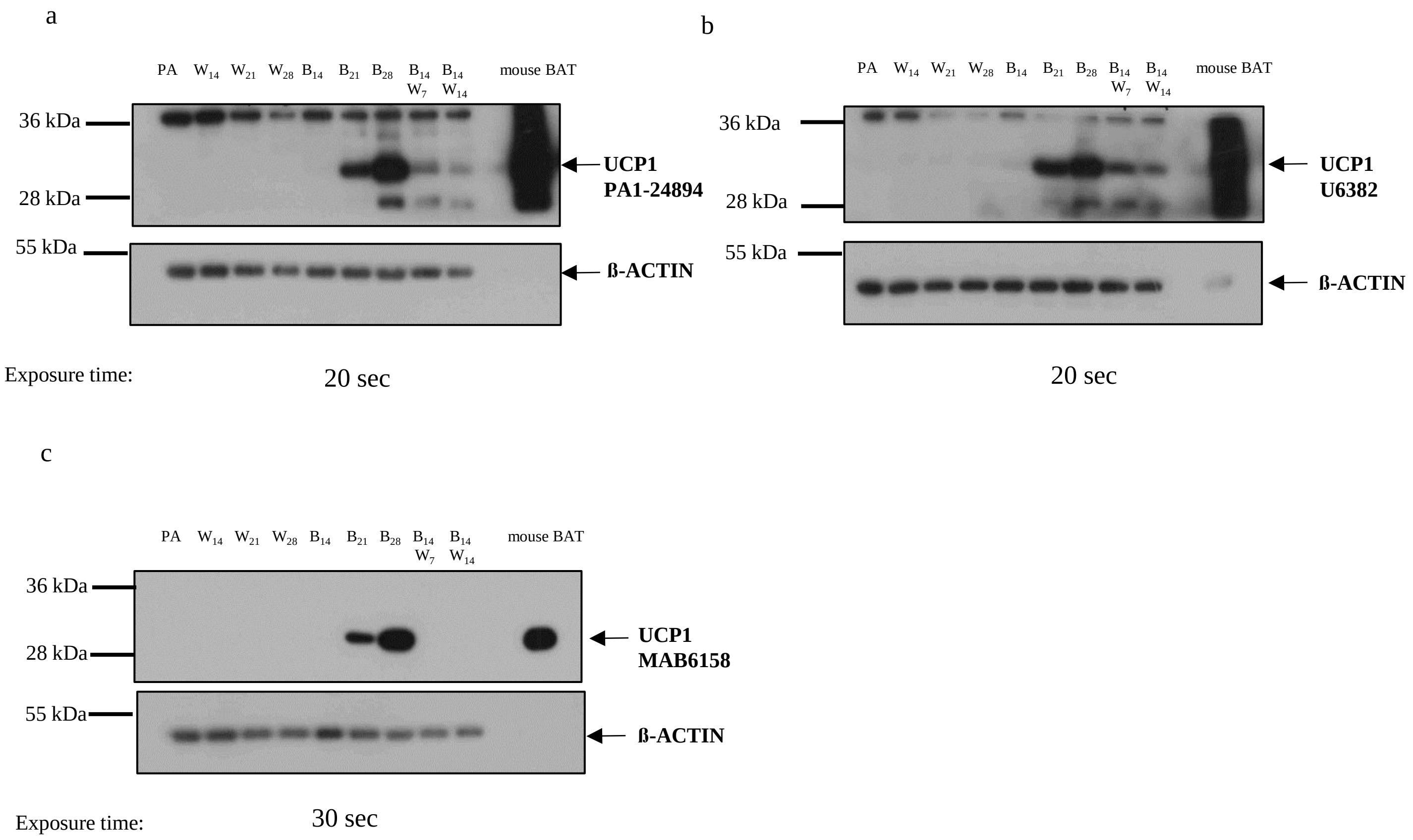

Supplementary Figure S8. Western blot analysis of UCP1 expression in one representative SGBS replicate determined by PA1-24894 polyclonal **(a)**, U6382 polyclonal **(b)** and MAB6158 monoclonal **(c)** anti-UCP1 antibody. ß-actin was used as endogenous control.
